# American Public Health Association

**Published:** 1894-09

**Authors:** Irving A. Watson

**Affiliations:** Secretary; Concord, N. H.


### AMERICAN PUBLIC HEALTH ASSOCIATION.

The American Public Health Association will convene at the city of Montreal, Canada, Tuesday, September 25th, 1894, at nine o'clock A. M., and continue four days.

The regular sessions will be in Association Hall, Y. M. C. A. Building, Dominion Square, opposite the Hotel Windsor. The following topics have been selected for consideration at this meeting: 1. The Pollution of Water Supplies; 2. The Disposal of Garbage and Refuse; 3. Animal Diseases and Animal Food; 4. The Nomenclature of Disease and Forms of Statistics; 5. Protective Inoculations in Infectious Diseases; 6. National Health Legislation; 7. The Cause and Prevention of Diphtheria; 8. Causes and Prevention of Infant Mortality; 9. The Restriction and Prevention of Tuberculosis; 10. Car Sanitation; 11. The Prevention of the Spread of Yellow Fever.

Upon all of the above subjects special committees have been appointed; therefore all papers upon these topics should be presented to the appropriate committee in season to be incorporated as a part of the report of the committee, if deemed advisable.

The Executive Committee announces the following additional subjects upon which papers are invited: 12. On the Education of the Young in the Principles of Hygiene; 13. Private Destruction of Household Garbage and Refuse; 14. Disinfection of Dwellings after Infectious Diseases; 15. Inspection of School Children with reference to the Eyesight.

Papers will be received on miscellaneous sanitary and hygienic subjects, but preference will be given to the topics announced above.

All communications relating to local matters should be addressed to Dr. Elzéar Pelletier, secretary local committee of arrangements, No. 76 St. Gabriel Street, Montreal, Canada.

The meetings of the Association are open to the public. All persons, of whatever profession or occupation, interested in the work of the Association, are cordially invited to be present. Ladies are especially invited to attend the evening meetings.

Per order,

IRVING A. WATSON,

CONCORD, N. H., August 24th, 1894. *Secretary.*

